# Purification and Characterization of a Novel Alginate Lyase from the Marine Bacterium *Bacillus* sp. Alg07

**DOI:** 10.3390/md16030086

**Published:** 2018-03-09

**Authors:** Peng Chen, Yueming Zhu, Yan Men, Yan Zeng, Yuanxia Sun

**Affiliations:** 1 National Engineering Laboratory for Industrial Enzymes, Tianjin Institute of Industrial Biotechnology, Chinese Academy of Sciences, Tianjin 300308, China; chen_p@tib.cas.cn (P.C.); zhu_ym@tib.cas.cn (Y.Z.); men_y@tib.cas.cn (Y.M.); zeng_y@tib.cas.cn (Y.Z.); 2 University of Chinese Academy of Sciences, Beijing 100049, China

**Keywords:** alginate lyase, marine bacterium, *Bacillus* sp. Alg07, purification, alginate oligosaccharides

## Abstract

Alginate oligosaccharides with different bioactivities can be prepared through the specific degradation of alginate by alginate lyases. Therefore, alginate lyases that can be used to degrade alginate under mild conditions have recently attracted public attention. Although various types of alginate lyases have been discovered and characterized, few can be used in industrial production. In this study, AlgA, a novel alginate lyase with high specific activity, was purified from the marine bacterium *Bacillus* sp. Alg07. AlgA had a molecular weight of approximately 60 kDa, an optimal temperature of 40 °C, and an optimal pH of 7.5. The activity of AlgA was dependent on sodium chloride and could be considerably enhanced by Mg^2+^ or Ca^2+^. Under optimal conditions, the activity of AlgA reached up to 8306.7 U/mg, which is the highest activity recorded for alginate lyases. Moreover, the enzyme was stable over a broad pH range (5.0–10.0), and its activity negligibly changed after 24 h of incubation at 40 °C. AlgA exhibited high activity and affinity toward poly-β-d-mannuronate (polyM). These characteristics suggested that AlgA is an endolytic polyM-specific alginate lyase (EC 4.2.2.3). The products of alginate and polyM degradation by AlgA were purified and identified through fast protein liquid chromatography and electrospray ionization mass spectrometry, which revealed that AlgA mainly produced disaccharides, trisaccharides, and tetrasaccharide from alginate and disaccharides and trisaccharides from polyM. Therefore, the novel lysate AlgA has potential applications in the production of mannuronic oligosaccharides and poly-α-l-guluronate blocks from alginate.

## 1. Introduction

Alginate is a linear copolymer that is composed of homopolymeric blocks of (1–4)-linked α-l-guluronic acid (G) and its C5 epimer β-d-mannuronic acid (M), which forms three types of blocks: poly-α-l-guluronate (polyG), poly-β-d-mannuronate (polyM), and random heteropolymeric sequences (polyMG) [1]. Alginate is the most abundant carbohydrate in brown algae, and it accounts for up to 10–45% of the dry weight of brown algae [2]. Some bacteria that belong to the genera *Azotobacter* [3] and *Pseudomonas* [4] produce alginate as an extracellular polysaccharide. In contrast to algal alginate, bacterial alginate is acetylated. Commercial alginate manufactured from brown algae has been used as a thickening agent or gelling agent in the food and pharmaceutical industries [5]. Alginate can be degraded into alginate oligosaccharides (AOS) through a chemical process or by alginate lyase. Given that AOS can stimulate the growth of endothelial cells [6] and the production of multiple cytokines [7], they may be applied as growth-promoting agents in some plants [8] and bifidobacteria [9]. Furthermore, AOS demonstrate excellent antioxidant activity [10] and havepotential uses in protection against pathogens [11].

In alginate degradation, alginate lyases cleave the (1–4)-linked glucosidic bond of alginate via a β-elimination mechanism and generate unsaturated oligosaccharides with 4-deoxy-alpha-l-erythrohex-4-enopyranuronosyl uronate as the nonreducing terminal residue [12]. Numerous alginate lyases have been isolated from various organisms, such as marine algae [13], marine mollusks [14], marine and terrestrial bacteria [15,16], marine fungi [17], and viruses [18]. Alginate lyases can be categorized into polyM-specific, polyG-specific, and polyMG-specific lyases on the basis of their substrate preferences [19] or into endo- or exo-alginate lyases on the basis of their cleavage mode [19]. In the carbohydrate-active enzyme database, alginate lyases belong to the polysaccharide lyase family [20]. The structures of some alginate lyases have been elucidated.

Alginate lyases are widely used in many fields. For example, alginate lyases have been employed to explain the fine structures of alginate [21] and to prepare red and brown algal protoplasts [22]. These enzymes may be utilized in the treatment of cystic fibrosis [23] and have been used as catalysts for AOS production [24]. The application of alginate lyases in alginate degradation under mild conditions has recently attracted public attention given the high efficiency and specifi of these enzymes. Nevertheless, present studies on alginate lyases remain in development, and the low catalytic efficiency and poor thermostability of alginate lyases limit their utility in AOS production. Therefore, high-efficiency and thermostable alginate lyases should be identified for use in AOS production.

In our work, we isolated and identified *Bacillus* sp. Alg07, a novel marine bacterium. AlgA, the alginate lyase secreted by this strain, showed extremely high activity. Hence, we purified and characterized AlgA to confirm that it has potential applications in AOS production.

## 2. Results and Discussion

### 2.1. Screening and Identification of Strain Alg07

Twenty-one strains with alginate lyase activity were isolated using alginate as the sole carbon source. Alg07 secreted the alginate lyase with the highest activity in the fermentation culture.

**Figure 1:**
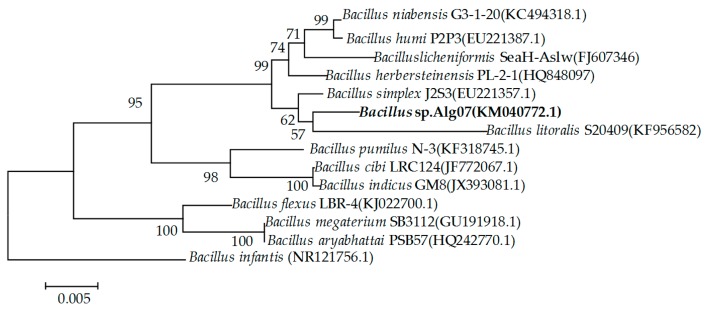
Neighbor-joining phylogenetic tree generated on the basis of the 16S rRNA gene sequences of strain Alg07 and other known *Bacillus* species.

The 16S rRNA gene of Alg07 was cloned, sequenced, and submitted to GeneBank (accession number KM040772) for strain identification. The alignment of 16S rRNA gene sequences from different *Bacillus* species showed that strain Alg07 is closely related to *Bacillus litoralis* S20409 (97%) and *Bacillus simplex* J2S3 (97%). However, the low similarity shared by the 16s rRNA gene sequence of Alg07 with that of other known *Bacillus* species indicated that Alg07 may be a novel *Bacillus* species. In accordance with the neighbor-joining phylogenetic tree, the strain was assigned to the genus *Bacillus* and designated as *Bacillus* sp. Alg07 (Figure 1).

### 2.2. Purification of Alginate Lyase from Bacillus sp. Alg07

*Bacillus* sp. Alg07 was cultured in optimized liquid medium for 24 h until its alginate lyase reached the highest activity. The supernatant containing 510 U/mL of alginate lyase was subjected to further purification through the two simple steps of tangential flow filtration concentration and anion exchange chromatography with Source 15Q (Figure S1). After purification, the alginate lyase was purified 8.34-fold with a yield of 62.4% (Table 1). The final specific activity of the purified alginate lyase was 8306.7 U/mg. The purified alginate lyase from *Bacillus* sp. Alg07 was designated as AlgA.

The activities of AlgA and those of other well-studied strains are shown in Table 2, which shows that AlgA has the highest activity among all reported alginate lyases. The simple purification, high recovery, and high specific enzyme activity of AlgA indicated that it may be produced industrially on a large scale.

**Figure 2:**
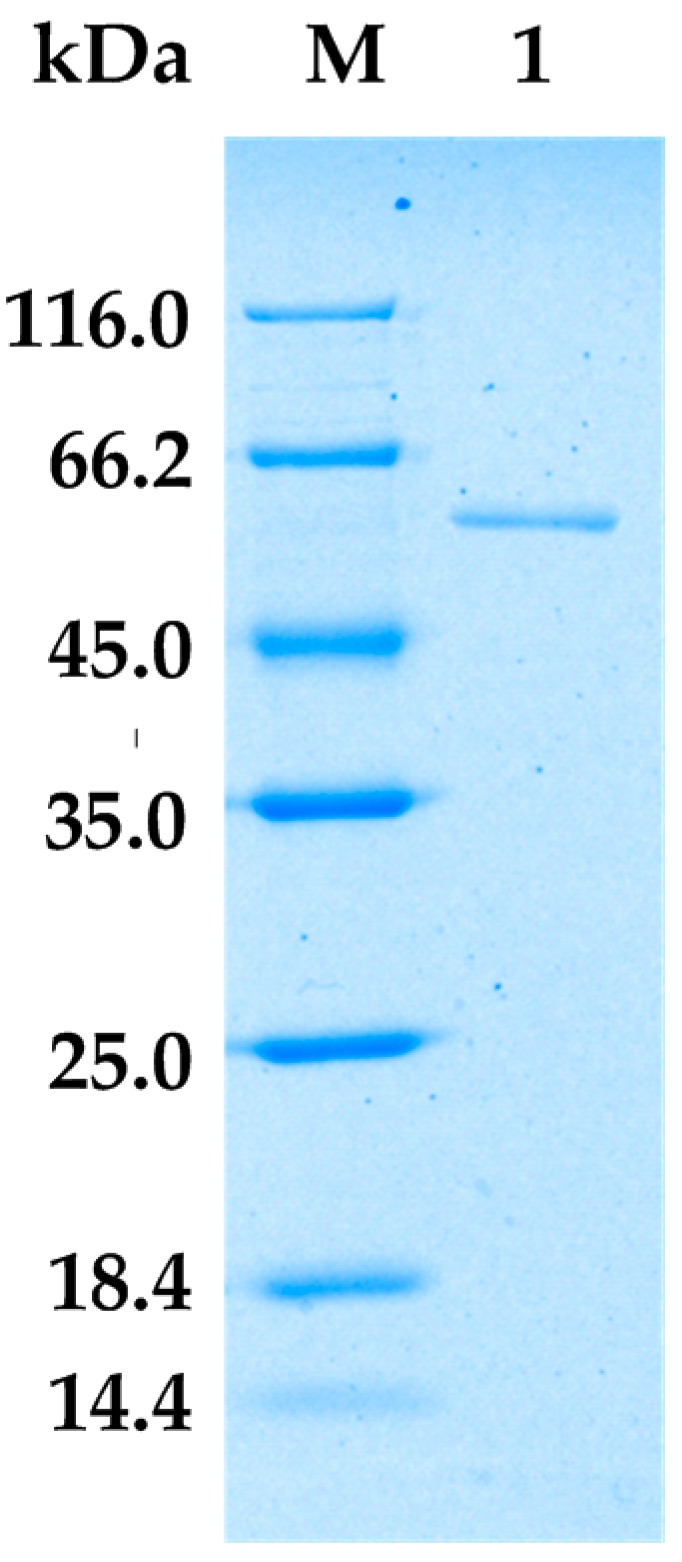
Sodium dodecyl sulfate polyacrylamide gel electrophoresis (SDS-PAGE) result for AlgA. Lane M, protein ladder; Lane 1, purified AlgA.

Figure 2 shows that the purified AlgA exhibited a clear and unique band on sodium dodecyl sulfate polyacrylamide gel electrophoresis (SDS-PAGE). This result suggested that the two-step purification process is successful. The molecular weight of AlgA was approximately 60 kDa, which is similar to molecular weights of alginate lyases from *Vibrio* sp.YKW-34 [30] and *Saccharophagus degradans* [26]. AlgA belongs to the class of large alginate lyases on the basis of its molecular weight [31].

The N-terminal amino acid sequence of the purified AlgA was analyzed. The sequence was identified as Glutamic acid–Glutamic acid–Glutamic acid–Glutamic acid–Aspartic acid–Valine–Threonine–Tyrosine (Figure S2). The results from homology search using BLASTp and CLUSTAL X indicated that the N-terminal amino acid sequence of AlgA is absent from the sequences of the previously reported alginate lyases. Furthermore, a protein with four Glu residues at its N-terminal is unusual. Therefore, AlgA may be a novel alginate lyase.

### 2.3. Biochemical Characterization of AlgA

**Figure 3:**
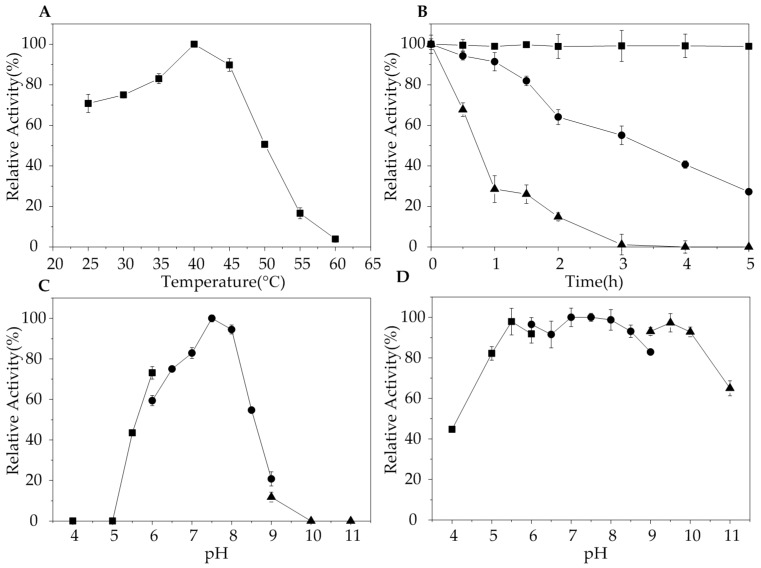
Effects of temperature and pH on the relative activity of AlgA. (**A**) Optimal temperature of AlgA. (**B**) Thermostability of AlgA at 40 °C (filled square), 45 °C (filled circle), and 50 °C (filled triangle). (**C**) Optimal pH for the relative activity of AlgA was determined in 20 mM CH_3_COOH-CH_3_COONa buffer (filled square), 20 mM Tris-HCl buffer (filled circle), or 20 mM Glycine-NaOH buffer (filled triangle). (**D**) pH stability of AlgA in 20 mM CH_3_COOH-CH_3_COONa buffer (filled square), 20 mM Tris-HCl (filled circle), and 20 mM Glycine-NaOH (filled triangle).

The optimal temperature of AlgA is 40 °C, which is similar to alginate lyases derived from *Vibrio* sp.YKW-34 [30], *Flavobacterium* sp. LXA [11], *Pseudomonas aeruginosa* PA1167 [32], and *Stenotrophomnas maltophilia* KJ-2 [33]. The activity of AlgA significantly decreased under temperatures exceeding 50 °C (Figure 3A). Thermostability analysis indicated that the activity of AlgA remained relatively unchanged after 5 h of incubation at 40 °C and did not decrease even after 24 h of incubation at 40 °C (data not shown). However, the enzyme was less stable under high temperatures, and the half-life of AlgA is approximately 3 h under 45 °C and 0.75 h under 50 °C (Figure 3B). This result indicated that at its optimal temperature, AlgA might be the most stable enzyme among all reported alginate lyases. Alginate lyases from *Favobacterium* sp. LXA [11], *Vibrio* sp. W13 [34], and *Zobellia galactanivorans* [35] are stable only under temperatures less than 40 °C. The activities of many alginate lyases considerably decrease after incubation at 40 °C. AlyL2 from *Agarivorans* sp. L11 has a half-life of 125 min at 40 °C [36], and OalS17 from *Shewanella* sp. Kz7 retains 88% of its activity after 1 h of incubation at 40 °C [25]. Furthermore, alginate lyases from *Flammeovirga* sp. MY04 [37] and *Vibrio* sp. SY08 [27] retain approximately 80 and 75%, respectively, of their activities after 2 h of incubation at 40 °C. Thermostable enzymes are more advantageous than thermolabile counterparts due totheir long half-lives and low production costs. Thus, AlgA has potential industrial applications given its excellent thermostability.

The optimal pH of AlgA was 7.5 in 20 mM Tris-HCl buffer. AlgA exhibited more than 90% of its maximal activity in pH 8.0 buffer. These results suggested that AlgA is basophilic (Figure 3C). Similarly, the optimal activities of most alginate lyases from marine bacteria are observed at pH 7.5–8.0 (Table 2). The results of the pH stability assay showed that AlgA presented the highest stability for an extended period over a pH range of 5.0–10.0 (Figure 3D). The good stability of AlgA over a wide pH range indicated its suitability for industrial application. Furthermore, the use of AlgA may decrease production costs given that pH adjustment will be unnecessary even when various alginates from different sources are employed as substrates.

**Figure 4:**
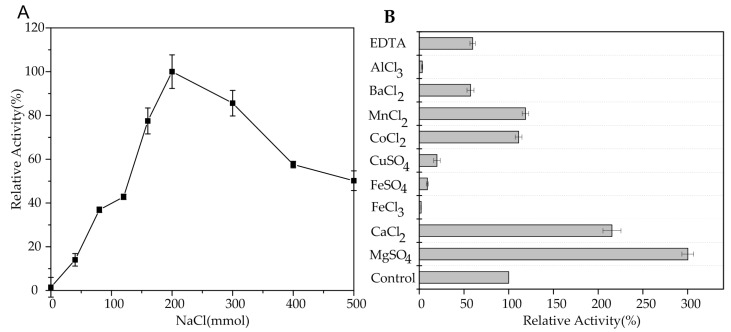
Effect of NaCl (**A**) and metal ions (**B**) on the activity of AlgA.

To determine the effect of NaCl on AlgA, the activity of AlgA in the presence of various NaCl concentrations was measured. As shown in Figure 4A, the optimal NaCl concentration for AlgA activity was 200 mM, and no activity was detected in the absence of NaCl. These results indicated that the activity of AlgA is dependent on NaCl. Thus, NaCl concentration is crucial for the activity of AlgA, which is a salt-activated alginate lyase. However, high NaCl concentrations decreased the activity of AlgA. Similarly, the activities of marine bacterial alginate lyases, such as AlyV5 from *Vibrio* sp. QY105 [28] and AlyYKW-34 from *Vibrio* sp. YKW-34 [30], are dependent on NaCl.

The effects of metal ions and EDTA on the activity of AlgA were determined in the presence of 200 mM NaCl. Mg^2+^ or Ca^2+^ significantly enhanced enzymatic activity by 300% or 215%, and Co^2+^ and Mn^2+^ slightly increased enzymatic activity (Figure 4B). By contrast, Hg^2+^, Fe^3+^, Fe^2+^, and Cu^2+^ completely inhibited lyase activity. Ba^2+^ and EDTA partially inhibited lyase activity. Ca^2+^ and Mg^2+^ increase the activity of many alginate lyases, such as aly-SJ02 from *Pseudoalteromonas* sp. SM0524 [38] and AlyV5 from *Vibrio* sp. QY105 [28]. However, Mg^2+^ and Ca^2+^ decrease the activity of Alg7D from *S. degradans* [26].

### 2.4. Substrate Specificity and Kinetic Parameters of AlgA

**Figure 5:**
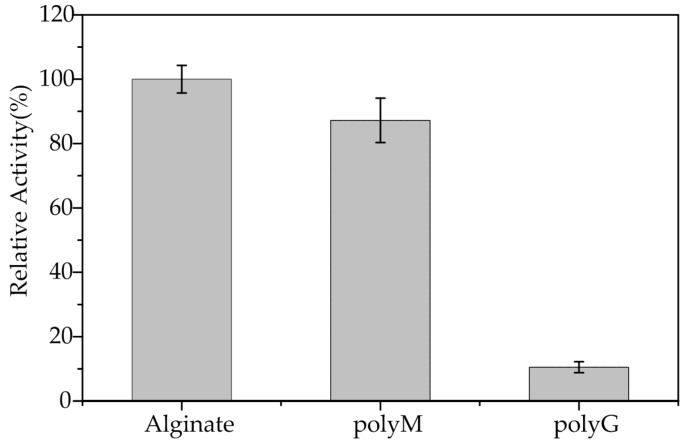
Relative activities of AlgA toward alginate, polyM, and polyG.

AlgA exhibited activity toward alginate but not toward pectin, hyaluronan, chitin, or agar. This behavior suggested that AlgA is indeed an alginate lyase. As shown in Figure 5, the relative activities of AlgA toward alginate, polyM, and polyG blocks are 100 ± 4.3, 87.2 ± 6.9, and 10.5 ± 1.7%, respectively. The slight activity toward polyG block might result from the presence of a few M residues in polyG substrates. These results indicated that the polyM block of substrates is the preferred substrate of AlgA. Thus, AlgA is a mannuronate lyase. Alginate lyases from *Flavobacterium* sp. UMI-01 [16], *Vibrio* sp. JAM-A9m [39], and *Pseudomonas* sp. QD03 [40] also belong to the mannuronate lyase class of enzymes.

The kinetic parameters of AlgA were determined through nonlinear regression analysis (Figure S3) and are shown in Table 3. AlgA has a lower *Km* value for polyM than for sodium alginate. This result suggested that AlgA has high affinity for polyM blocks and further confirmed that AlgA is a mannuronate lyase. However, the *kcat* values of AlgA for sodium alginate were higher than those for polyM. Therefore, AlgA has equivalent catalytic efficiency for sodium alginate and polyM.

### 2.5. Fast Protein Liquid Chromatography and Electrospray Ionization Mass Spectrometry Analysis of the Degradation Products of AlgA

**Figure 6:**
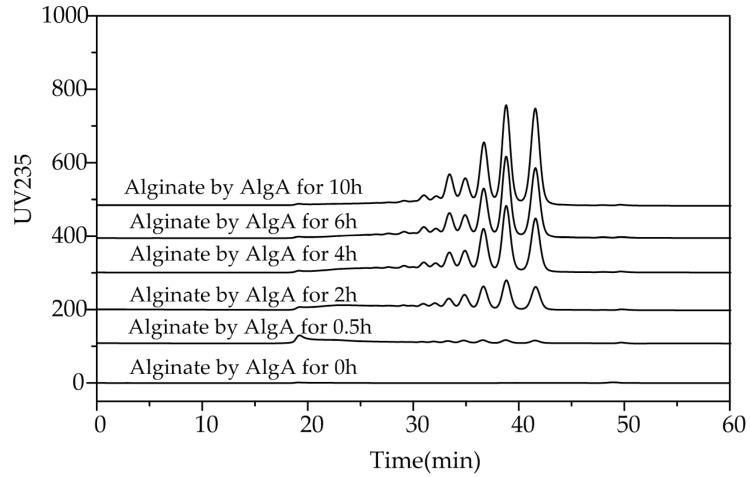
Patterns of the polysaccharide degradation products of AlgA. Enzymatic degradation products collected at 0.5, 2, 4, 6, and 10 h were subjected to gel filtration with a Superdex peptide 10/300 GL column. The absorbances of the products were monitored at 235 nm.

To investigate the action patterns of AlgA, degradation of alginate by this enzyme was performed. The degradation products at different time intervals were analyzed through gel chromatography. Fast protein liquid chromatography (FPLC) analysis indicated that AOS with different degrees of polymerization (DP) gradually accumulated (Figure 6). Therefore, AlgA is an endo-type alginate lyase.

**Figure 7:**
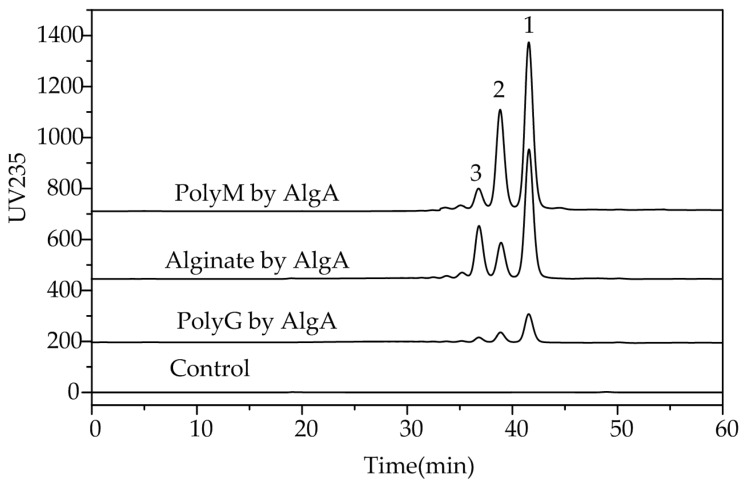
Final products of alginate, polyM, and polyG after degradation by AlgA. Oligosaccharide products were gel-filtered through a Superdex peptide 10/300 GL column and monitored at a wavelength of 235 nm.

In addition, the alginate was completely digested with an excess of AlgA at 40 °C for 24 h. The products were separated through gel chromatography. The elution profiles (Figure 7) of the degradation products presented three major fractions (peaks 1, 2, and 3).

**Figure 8:**
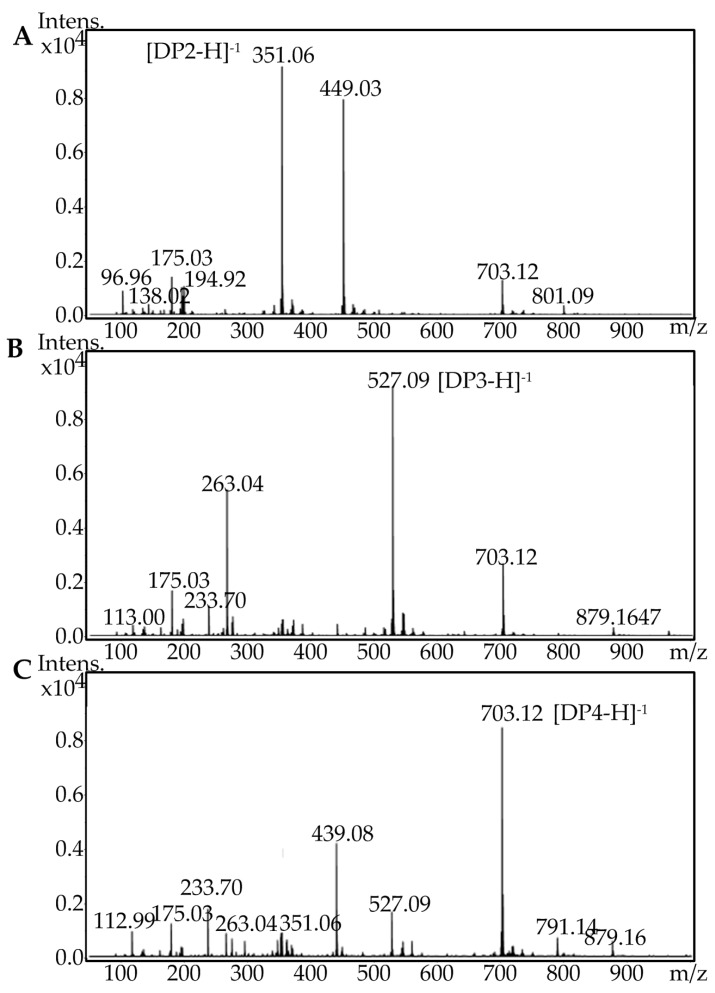
Electrospray ionization mass spectrometry (ESI-MS) analysis of the final oligosaccharide products. (**A**) Fraction peak 1 separated through fast protein liquid chromatography (FPLC), (**B**) fraction peak 2 separated through FPLC, and (**C**) fraction peak 3 separated through FPLC.

To identify the final oligosaccharide products of AlgA degradation and to determine their DP, three major fractions were subjected to electrospray ionization mass spectrometry (ESI-MS). The molecular masses of oligosaccharides in peaks 1, 2, and 3 were determined to be 351.06, 527.09, and 703.12, respectively (Figure 8). These results indicated that the main degradation products are di-, tri- and tetra-saccharides. The relative contents of di-, tri- and tetra-saccharides in alginate were 61.19%, 15.59%, and 23.22%, respectively, whereas those of di-, tri- and tetra-saccharides in the polyM substrate were 58.30%, 34.26%, and 7.43%, respectively. Meanwhile, AlgA has limited activity toward polyG. Therefore, AlgA may be used in the production of mannuronic oligosaccharides from polyM blocks and the preparation of polyG blocks from sodium alginate via the degradation of polyM blocks. The products of mannuronic oligosaccharides and polyG blocks possess special biological activity and have potential applications in many fields. For example, mannuronate oligosaccharides can promote the secretion of multiple cytokines [7], and polyG demonstrates higher macrophage-stimulation activity than polyM [41].

## 3. Materials and Methods

### 3.1. Materials

Sodium alginate derived from brown seaweed was purchased from Sigma (St. Louis, MO, USA). SOURCE^TM^ 15Q 4.6/100 PE and Superdex peptide 10/300 gel filtration columns were purchased from GE HealthCare Bio-Sciences (Uppsala, Sweden). DNA polymerase, protein molecular weight markers, and polyacrylamide were purchased from New England Biolabs (Ipswich, MA, USA). Other chemicals and reagents used in this study were of analytical grade.

### 3.2. Screening and Identification of Strain Alg07

Sea mud and rotten kelp samples were collected from a seaweed farm in Weihai, China. Five grams of the samples were added to 45 mL of modified marine broth 2216 medium containing 5 g/L (NH_4_)_2_SO_4_, 19.45 g/L NaCl, 12.6 g/L MgCl_2_·6H_2_O, 6.64 g/L MgSO_4_·7H_2_O, 0.55 g/L KCl, 0.16 g/L NaHCO_3_, 1 g/L ferric citrate, and 10 g/L sodium alginate. After 48 h of enrichment at 30 °C, the culture was serially diluted with deionized water and spread on modified marine broth 2216 agar containing 10 g/L sodium alginate. The plates were incubated at 30 °C for two days until colonies appeared. Single colonies were inoculated into marine broth 2216 medium containing 10 g/L sodium alginate and incubated for 48 h at 30 °C. Then, the activities of alginate lyase in the supernatants were determined. The alginate lyase from one of the isolates, strain Alg07, showed the highest activity among all lyases.

To identify the Alg07 strain, the 16S rRNA gene of the strain was amplified through PCR by using universal primers. The purified PCR fragment was sequenced and compared with reported 16s rRNA sequences in Genbank by using BLAST. A phylogenetic tree was constructed using CLUSTAL X and MEGA 6.0 [42] through neighbor-joining method [43].

### 3.3. Production and Purification of AlgA

Strain Alg07 was cultured for 24 h at 30 °C and 180 rpm in the optimized liquid medium, which contained 1g/L peptone, 3 g/L yeast extract, 9 g/L sodium alginate, 5 g/L NaCl, 1 g/L MgSO_4_·7H_2_O, 5 g/L KCl, and 4 g/L CaCl_2_ (pH 6.5). The supernatant was collected after 30 min of centrifugation at 10,000 rpm and 4 °C and then concentrated and desalted using a tangential flow filtration system (Vivaflow 50, Sartorius, Goettingen, Germany). The concentrated solution was subjected to AKTA FPLC (GE Healthcare Life Science, Marlborough, MA, USA) equipped with a SOURCE^TM^ (Barrie, ON, Canada) 15Q 4.6/100 PE column that had been equilibrated with 20 mM Tris–HCl buffer (pH 7.0). Adsorbed proteins were eluted with a linear gradient of 0–0.5 M NaCl in equilibrating buffer under a flow rate of 1 mL/min. Fractions possessing the highest specific activity among all fractions were pooled and dialyzed against 20 mM Tris-HCl buffer (pH 7.0) for further enzyme characterization. All purification procedures were performed at 4 °C. Protein concentration was determined with a protein quantitative analysis kit (Solarbio, Beijing, China) using bovine serum albumin as the calibration standard. The purity of the isolated alginate lyase was analyzed through 12.5% SDS-PAGE in accordance with the method of Laemmli (1970) [44].

### 3.4. Enzyme Activity Assay

To determine the activity of alginate lyase, 100 μL of appropriately diluted enzyme was added to 1900 μL of substrate solution containing 10 g/L sodium alginate, 20 mM Tris-HCl, and 200 mM NaCl (pH 7.5). The reaction was allowed to proceed for 20 min at 40 °C and terminated by the addition of 20 μL of 10 M NaOH. Absorbance at 235 nm was recorded. One unit (U) was defined as the amount of enzyme required to increase the absorbance at 235 nm by 0.1 per min. For kinetic parameter analysis, the 3,5-dinitrosalicylic acid method was performed to determine alginate lyase activity based on the release of reducing sugars from substrates [45]. One unit (U) was defined as the amount of enzyme required to release 1 μmol of reducing sugar per min. All enzyme reactions were performed in triplicate, and reaction parameters were expressed as mean ± standard deviation.

### 3.5. Characterization of AlgA

Enzyme reactions were carried out at different temperatures (25 °C–60 °C) to determine the effect of temperature on AlgA. To evaluate the thermal stability of AlgA, the enzyme was incubated for different intervals at 40 °C, 45 °C, and 50 °C. Then, the residual activities of the enzyme were tested. The activity of the enzyme stored at 4 °C was used to represent 100% enzyme activity. The effect of different pH values on AlgA was determined by calculating the residual activities of AlgA after 20 h of incubation at 4 °C in 20 mM sodium acetate (pH 3.0–6.0), Tris-HCl (pH 6.0–9.0), or glycine-NaOH (pH 9.0–11.0) buffers. The initial activities in different pH buffers represented 100% enzyme activity.

Enzyme reactions were performed in the presence of different concentrations of NaCl (0–500 mM) to evaluate the effect of NaCl on AlgA. To determine the effects of metal ions and EDTA on the activity of AlgA, the highest enzyme activity was considered as 100% enzyme activity. AlgA was subjected to an activity assay after 12 h of incubation at 4 °C in the presence of 2 mM of different metal ions and EDTA.

### 3.6. Analysis of the N-Terminal Amino Acid Sequence of AlgA

After SDS-PAGE, the protein band of AlgA was electro-transferred onto a polyvinylidene difluoride membrane (Imobulon; Millipore, Darmstadt, Germany). Amino acid sequences were determined with a PPSQ-31A protein sequencer (Shimadzu Corporation; Kyoto, Japan).

### 3.7. Substrate Specificity and Kinetic Parameters of AlgA

A standard enzymatic assay was performed to test the substrate preference of AlgA by using pectin, hyaluronan, chitin, agar, sodium alginate, polyM, and polyG as substrates. PolyM and polyG were prepared in accordance with the method of Haug et al. [46].

The kinetic parameters of AlgA toward alginate and polyM were determined by measuring the initial velocities of enzyme activity under various substrate concentrations and were calculated on the basis of the nonlinear regression fitting of the Michaelis–Menten equation using Prism 6 (GraphPad Software, Inc., La Jolla, CA, USA).

### 3.8. FPLC and ESI-MS Analysis of the Degradation Products of AlgA

To elucidate the mode of action of AlgA toward alginate, alginate degradation was performed at 40 °C with 10 g/L sodium alginate as a substrate. The reaction was initiated by the addition of 2 μg of purified AlgA in a 10 mL reaction volume. Reaction solutions were withdrawn at appropriate time intervals, and AlgA was inactivated by 5 min of boiling. The samples were analyzed through FPLC with a Superdex peptide 10/300 gel filtration column (GE Health, Marlborough, MA, USA) with 0.2 M ammonium bicarbonate as the mobile phase at a flow rate of 0.4 mL/min [34]. The reaction was monitored at 235 nm.

To determine the oligosaccharide compositions of the final digests, an excess of AlgA was used to completely degrade 10 g/L sodium alginate, polyM, or polyG. The reaction was carried out in a 10 mL reaction volume at 40 °C for 24 h and then terminated by boiling for 5 min. Oligosaccharides were separated through gel filtration as described above. Peak fractions containing unsaturated oligosaccharide products were collected and repeatedly freeze-dried to remove NH_4_HCO_3_ for ESI-MS analysis. The molecular weight of each oligosaccharide fraction was determined using the ESI-MS method on microTOF-Q II equipment (Bruker, Billerica, MA, USA) with the following conditions: capillary voltage of 4 kV, dry temperature of 180 °C, gas flow rate of 4.0 L/min, and scan range of 50–1000 *m*/*z*.

## 4. Conclusions

An alginate lyase–producing marine bacterium was isolated and identified as *Bacillus* sp. Alg07. AlgA, the alginate lyase derived from *Bacillus* sp. Alg07, was purified through the two simple steps of tangential flow filtration concentration and anion-exchange chromatography. The results of SDS-PAGE indicated that the molecular mass of AlgA is approximately 60 kDa. The optimal temperature and pH for the activity of AlgA is 40 °C and 7.5, respectively. The activity of AlgA is dependent on NaCl and is promoted by the addition of Mg^2+^ and Ca^2+^. Under optimal conditions, the specific activity of AlgA reaches up to 8306.7 U/mg, which is the highest activity recorded for all reported alginate lyases. Moreover, AlgA is stable over a broad pH range (5.0–10.0) and under its optimal temperature (40 °C). AlgA is an endolytic polyM-specific alginate lyase and mainly produces disaccharides, trisaccharides, and tetrasaccharides from alginate and disaccharides and trisaccharides from polyM. The highly efficient and thermostable AlgA can have potential applications in the production of mannuronic oligosaccharides and polyG blocks from alginate.

## Figures and Tables

**Table 1:** Summary of the purification of AlgA.

Step	Total Protein (mg)	Total Activity (U)	Specific Activity (U/mg)	Folds	Yield (%)
Culture broth	23.82	23,724.72	996.01	1.00	100
Vivaflow50	10.30	22,672.14	2201.18	2.21	95.4
Source 15Q	1.78	14,785.9	8306.71	8.34	62.4

**Table 2:** Comparison of the properties of AlgA with those of alginate lyases from different microorganisms.

Enzyme	Source	Specific Activity (U/mg)	Molecular Mass (kDa)	Optimal Temperature (°C)	Optimal pH	Cation Activators	Substrate Specificity
AlgA	This study	8306.71	60	40	7.5	Na^+^, Mg^2+^, Ca^2+^, Mn^2+^	PM
Oal17	*Shewanella* sp.Kz7 [25]	32	82	50	6.2	Na^+^	PM
Cel32	*Cellulophaga* sp. NJ-1 [15]	2417.8	32	50	8.0	Ca^2+^, Mg^2+^, K^+^	PM, PG
Alg7D	*Saccharophagus* degradans [26]	4.6	63.2	50	7.0	Na^+^	PM, PG
AlySY08	*Vibrio* sp. Aly08 [27]	1070.2	33	40	7.6	Na^+^, K^+^, Ca^2+^, Mg^2+^	PM, PG
AlyV5	*Vibrio* sp. QY105 [28]	2152	37	38	7.0	Na^+^, Mg^2+^, Ca^2+^, Mn^2+^	PM, PG
Alm	*Agarivorans* sp. JAM-Alm [29]	108.5	31	30	10.0	Na^+^, K^+^	PG, PMG
FlAlyA	*Flavabacterium* sp. UMI-01 [16]	2347.8	30	55	7.7	Na^+^, K^+^, Ca^2+^, Mg^2+^	PM

**Table 3:** Kinetic parameters of the activity of AlgA toward sodium alginate and polyM blocks.

Parameter	Sodium Alginate	PolyM
*Vmax* (U mg of protein^−1^)	1052.0 ± 214.6	547.6 ± 22.4
*Km* (mg mL^−1^)	9.0 ± 3.3	3.6 ± 0.4
*kcat* (s^−1^)	911.7 ± 185.9	474.6 ± 19.4
*kcat/Km* (mg^−1^ mL s^−1^)	101.3 ± 20.7	132.0 ± 5.4

## Supplementary Materials

The following are available online at http://www.mdpi.com/1660-3397/16/3/86/s1, Figure S1: Purification of alginate lyase by anion exchange chromatograph (Source 15Q); Figure S2. The N-terminal amino acid sequence of the purified AlgA; Figure S3. Effects of substrate concentration on the activity of AlgA.
