# Supplementary material for: Purification and Characterization of a Novel Alginate Lyase from the Marine Bacterium Bacillus sp. Alg07
Source: Mar Drugs. 2018 Mar 9;16(3):86. doi: 10.3390/md16030086 (PMC5867630; doi:10.3390/md16030086)
Supplement: Supplementary file 1 [file marinedrugs-16-00086-s001.pdf]

Supplementary Material

Purification and Characterization of a Novel Alginate Lyase from Marine Bacterium *Bacillus* sp.Alg07

Peng Chen [1,2], Yueming Zhu [1], Yan Men [1], Yan Zeng [1] and Yuanxia Sun [1,*]

[1] National Engineering Laboratory for Industrial Enzymes, Tianjin Institute of Industrial Biotechnology, Chinese Academy of Sciences, Tianjin 300308, China; chen_p@tib.cas.cn
[2] University of Chinese Academy of Sciences, Beijing 100049, China
* Correspondence: syx0430@hotmail.com; Tel.: +86-022-8486-1960

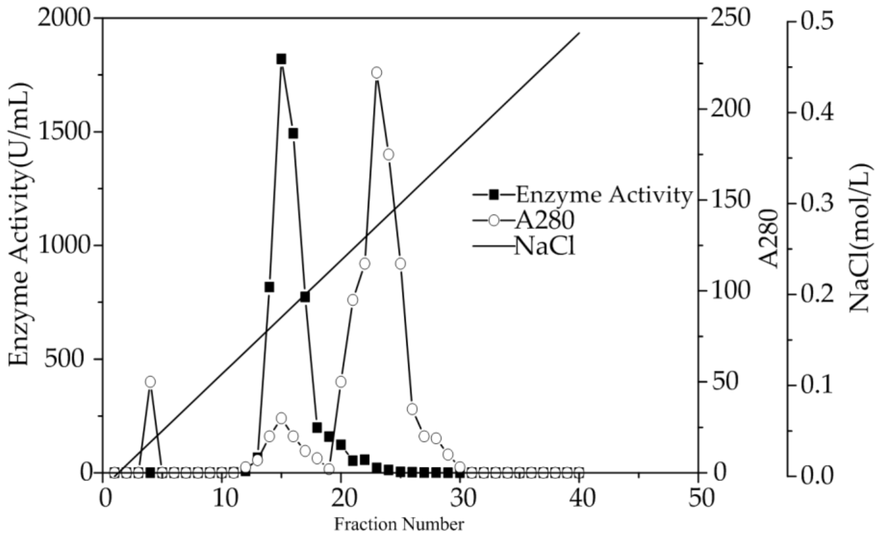

Figure S1 Purification of alginate lyase by anion exchange chromatograph (Source 15Q)

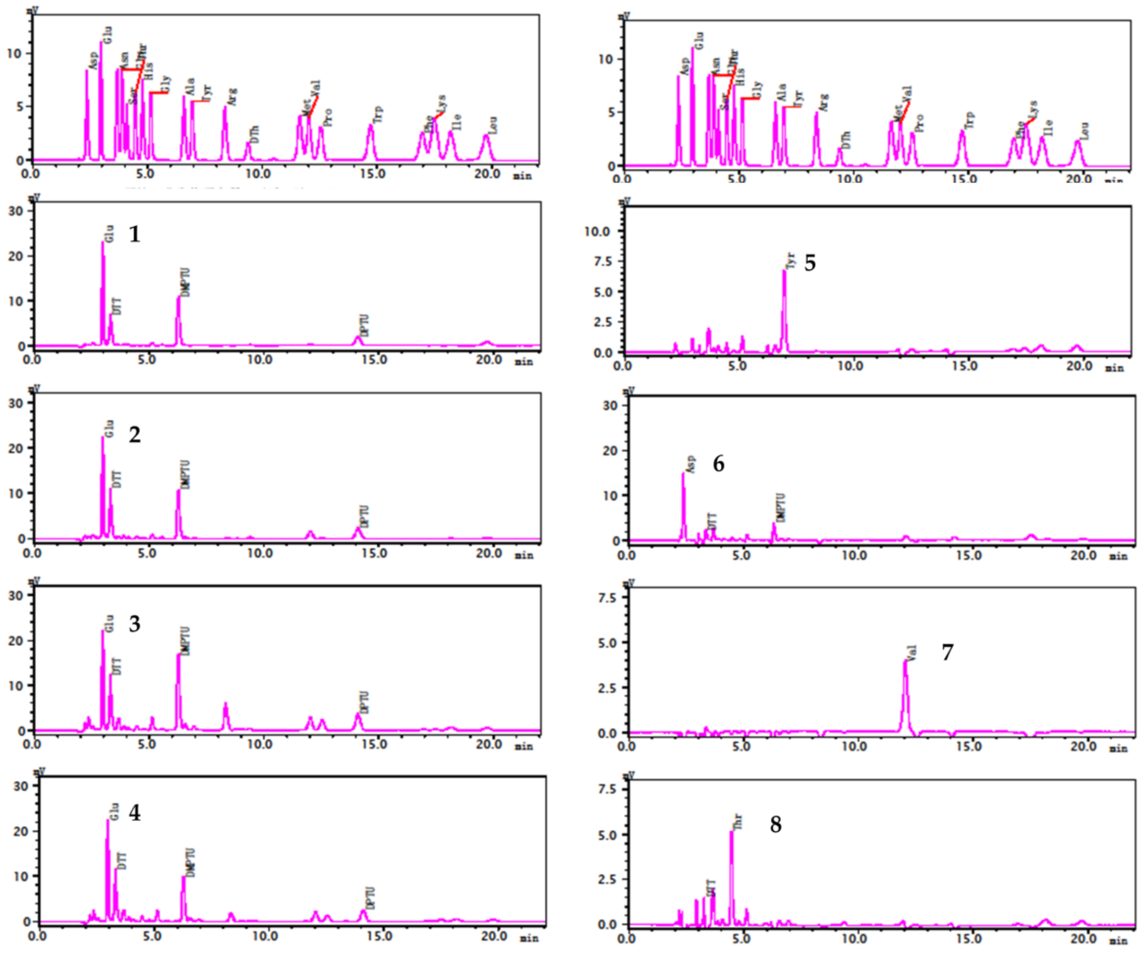

Figure S2 The N-terminal amino acid sequence of the purified AlgA

1: Glu; 2: Glu; 3: Glu; 4:Glu; 5: Tyr; 6: Asp; 7: Val; 8: Thr.

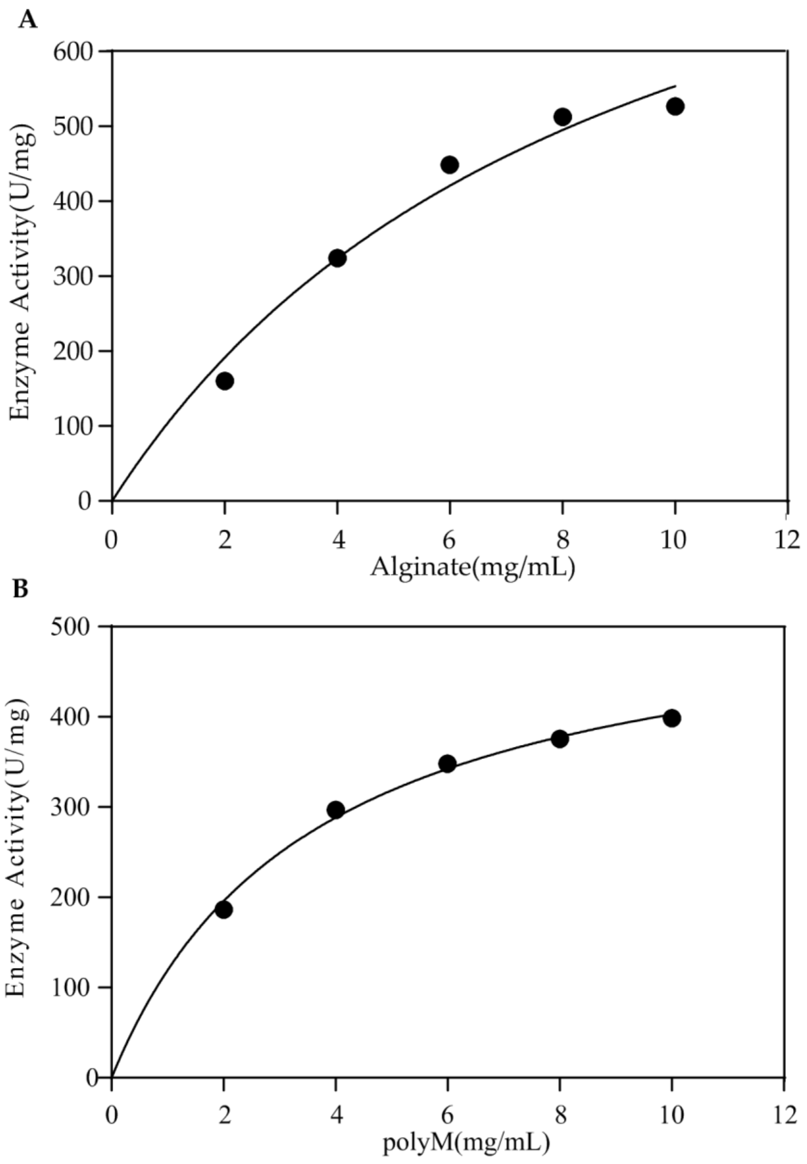

Figure S3 Effects of substrate concentration on the activity of AlgA. The activity of the enzyme was measured in the presence of indicated concentrations of substrates (A) sodium alginate, (B) PolyM.
